# Social support networks and life cycle stage of Venezuelan immigrant families

**DOI:** 10.1590/0034-7167-2022-0790

**Published:** 2023-12-08

**Authors:** Marcela Possato Correa da Rosa, Gisele Cristina Manfrini, Janaína Medeiros de Souza, Ana Cristina Oliveira da Silva Hoffmann, Rosane Goncalves Nitschke, Ivonete Teresinha Schülter Buss Heidemann, Jaime Alonso Caravaca-Morera

**Affiliations:** I Universidade Federal de Santa Catarina. Florianópolis, Santa Catarina, Brazil; II Universidad de Costa Rica. San José, Montes de Oca, Costa Rica

**Keywords:** Social Network Analysis, Life Cycle Stages, Family Nursing, Social Determinants of Health, Forced Displacement, Análisis de Redes Sociales, Estadios del Ciclo de Vida, Enfermería de la Familia, Determinantes Sociales de la Salud, Desplazamientos Forzados, Análise de Rede Social, Estágios do Ciclo de Vida, Enfermagem Familiar, Determinantes Sociais da Saúde, Deslocamento Forçado

## Abstract

**Objective:**

To understand the structures of social networks of interiorized Venezuelan immigrant families and the life cycle stage they are in.

**Methods:**

Qualitative multiple-case study with families from the Interiorization Program residing in the Greater Florianópolis, Brazil. Screening occurred through social networks and key informants. For data collection, the photovoice technique and semi-structured interviews were used, categorized with the help of the Atlas.ti software. Genograms and ecomaps were elaborated.

**Results:**

Of the 4 families interviewed, totaling 7 members with young children, the nuclear family was identified as the main informal support network. Formal networks included schools, churches, and civil society.

**Conclusion:**

Families rely heavily on their nuclear structure for support, with formal institutions acting as secondary resources. The predominant life cycle stage is focused on families with young children. More efforts are needed to strengthen formal support networks.

## RESUMO

**Objetivo:**

Compreender as estruturas das redes sociais de famílias imigrantes venezuelanas interiorizadas e a fase do ciclo vital em que se encontram.

**Métodos:**

Estudo qualitativo de múltiplos casos com famílias do Programa de Interiorização residentes na Grande Florianópolis, Brasil. A triagem ocorreu por meio de redes sociais e informantes-chave. Para a coleta de dados, utilizou-se a técnica de *photovoice* e entrevistas semiestruturadas, categorizadas com a ajuda do *software Atlas.ti*. Foram elaborados genogramas e ecomapas.

**Resultados:**

Das 4 famílias entrevistadas, totalizando 7 membros com filhos pequenos, identificou-se a família nuclear como principal rede de apoio informal. As redes formais incluíram escolas, igrejas e sociedade civil.

**Considerações finais:**

As famílias dependem fortemente de sua estrutura nuclear para apoio, com instituições formais atuando como recursos secundários. A fase do ciclo vital predominante está focada em famílias com filhos pequenos. Mais esforços são necessários para fortalecer as redes de apoio formais.

## RESUMEN

**Objetivo:**

Comprender las estructuras de las redes sociales de familias inmigrantes venezolanas interiorizadas y la fase del ciclo de vida en la que se encuentran.

**Métodos:**

Estudio cualitativo de múltiples casos con familias del Programa de Interiorización residentes en la Gran Florianópolis, Brasil. La selección se realizó a través de redes sociales e informantes clave. Para la recopilación de datos, se utilizó la técnica de fotovoz y entrevistas semiestructuradas, categorizadas con la ayuda del software Atlas.ti. Se elaboraron genogramas y ecomapas.

**Resultados:**

De las 4 familias entrevistadas, totalizando 7 miembros con hijos pequeños, se identificó a la familia nuclear como principal red de apoyo informal. Las redes formales incluyeron escuelas, iglesias y sociedad civil.

**Conclusiones:**

Las familias dependen en gran medida de su estructura nuclear para el apoyo, con las instituciones formales actuando como recursos secundarios. La fase predominante del ciclo de vida se centra en familias con hijos pequeños. Se requieren más esfuerzos para fortalecer las redes de apoyo formales.

## INTRODUCTION

According to the latest annual report of the United Nations Refugee Agency, Global Trends (2022), 100 million people were forcibly displaced worldwide. These data did not yet account for people displaced from Afghanistan after the United States’ withdrawal or from Ukraine with the start of the war^(1)^.

In Latin America, due to the political, economic, and humanitarian crisis, the migration of Venezuelans amounts to a total of 4 million people, being considered the largest exodus in the history of the region^(2)^.

In Brazil, it is known that the Venezuelan population has the state of Roraima as its main gateway ^(3, 4)^. After crossing the border, many newcomers do not find a support network on Brazilian soil, a situation that highlights the unpreparedness to receive the large contingent of people who arrive in a short period of time^(3, 4, 5)^. Since 2018, the Brazilian government and international agencies have started Operation “Acolhida” with the aim of providing border ordering, shelter, and interiorization^(6)^.

The Interiorization Program is aimed at immigrants who, voluntarily, want to move from the state of Roraima to other Brazilian states. For this, air tickets are provided to individuals and their families who fall into one of the program’s categories, which are: institutional (shelter-to-shelter), social reunion, family reunion, and with a signaled job vacancy^(6)^. Since 2018, approximately 96,000 immigrants have been interiorized, of which 88% traveled in family groups, with the state of Santa Catarina receiving the most interiorized Venezuelans^(7)^.

The presence of new migratory flows to Brazil has raised questions about human rights, with access to health and the integration of this population in society and the labor market identified as problems to be studied^(8)^. The migratory process involves different health risks for people, and in the family context, migration can be considered a non-normative process (unexpected in the life cycle), permeated by situations of stress, violence, or persecution, in which the person or group migrates in an unplanned way^(9, 10)^.

According to Sharon Denham, the life and health of families are affected by dynamic relationships between several systems, which include from the family and neighborhood system (microsystem) to the cultural and political system (macrosystem)^(11)^.

Within this perspective, the social support network can be defined as the sum of the relationships that an individual perceives as significant and constitutes an important element in their experience of identity, well-being, care habits, health, and adaptation capacity in a crisis^(12)^.

In nursing, the social support network is a key element for assessment in care, seeking to understand the life context and to know the resources to be mobilized in the moments of the life cycle and health-disease of individuals and families^(13)^.

## OBJECTIVE

To understand the structures of social networks of Venezuelan immigrant families who have been interiorized and the life cycle stage they are in.

## METHODS

### Ethical aspects

The study was conducted in accordance with national and international ethical guidelines and approved by the Research Ethics Committee with Human Beings at the Federal University of Santa Catarina, whose opinion is attached to the present submission. Free and Informed Consent was obtained from all individuals involved in the study in writing and translated into Spanish.

The participants received codes according to the identification number of each family: F1/F2/F3/F4 and of the members who participated in the interviews: (G) grandmother, (D) daughter, (M) mother, (P) father. (AF1) = aunt of family 1.

### Theoretical-methodological framework

For this study, the theoretical framework of Family Health Routines and Rituals by Sharon Denham was used. According to the author, social support networks can be divided into informal networks (family, friends, neighbors) and formal networks (health services, social assistance, education, and churches)^(11)^.

### Study type

This is a qualitative study, with a multiple case study design and narrative analysis, using the Consolidated Criteria for Reporting Qualitative Research (COREQ) instrument as a guide for describing the methodological procedures.

### Study setting

The study sites were the cities of Greater Florianópolis: Biguaçu, Florianópolis, and São José, in the state of Santa Catarina. The choice of location occurred because the state is the federative unit with the largest number of interiorized people since the beginning of the operation^(7)^.

### Sample

The study population consisted of immigrant nuclei of Venezuelan nationality, over the age of 18, who participated in the Interiorization Program of the Operation Acolhida. Venezuelan families who did not participate in the Interiorization Strategy were excluded from the study.

Although the Venezuelan migratory flow is considered a forced migratory flow, due to the crisis conditions in which it occurs, it is mentioned as a mixed flow, where there are people seeking asylum (with a request still pending), recognized refugees, temporary and permanent residents, among others. For this reason, we chose not to consider the migratory status of the research participants^(1)^.

The selection of participants took place through active search on social networks, in profiles and communities with the aim of bringing together Venezuelans, as well as by the dissemination of online calls also via social networks.

### Data collection and organization

Based on three initial key informants, found on social networks, other families that participated in the Interiorization Process were indicated. Of the total of 12 families indicated by the key informants, five initially agreed to participate in the research, but one withdrew due to lack of time for interviews.

Data collection took place between April and November 2021, at the homes of the families. The photovoice^(14)^ and semi-structured interview techniques were used, divided into two moments: in the first, data collection through images (photos); in the second, semi-structured interviews were conducted in the homes of each family.

Precautionary care was observed due to the pandemic period, with conversations in open and ventilated environments, use of masks and alcohol gel. For photographic records, the activity was guided by the guiding question: “What routines of your family represent the care of your health?” The interviews, lasting 30 to 50 minutes, were audio-recorded and subsequently transcribed and translated into Spanish, in order to prioritize the rigor in the validation of the data with the migrants. After collecting the data from genograms and ecomaps of each family, the diagrams were created using the GenoPro software.

### Data analysis

The data analysis followed the steps of Thematic Analysis. The data organization and categorization process were assisted by the Atlas.ti software. The categories were described and discussed in the light of the literature. From the analyzed data, four categories were distinguished.

## RESULTS

### Family Composition and Phase of the Family Cycle

From the data on the family structure of four Venezuelan immigrant nuclei, comprising six adults, aspects related to support networks, family composition, educational background, and developmental stage were identified. The family units consisted of three heterosexual couples; in one of them, the wife lived in Santa Catarina while the husband still resided in Manaus. One of the nuclei was female-headed. Five women, aged between 25 and 50, and one 32-year-old man were interviewed. All participating families in the study had children aged between 0 and 11, totaling 7 children.

The total migration time averaged five years since arriving in Brazil and ranged from 2 years to 1 month to reach Santa Catarina. This duration only considers the family members who arrived through the interiorization process, excluding those who came on their own, as in the case of the mother from family 1 (MF1).

Regarding the level of education: in the first family, the aunt had higher education and postgraduate studies; the mother had higher education; and the grandmother had completed high school. In the second family, both parents had incomplete higher education. In the third family, both parents had completed high school, and in the fourth family, the mother pursued technical education. None of the interviewees worked in their field of study, even with experience. In all cases, participants expressed dissatisfaction for not working in their respective fields.

**Chart 1:**
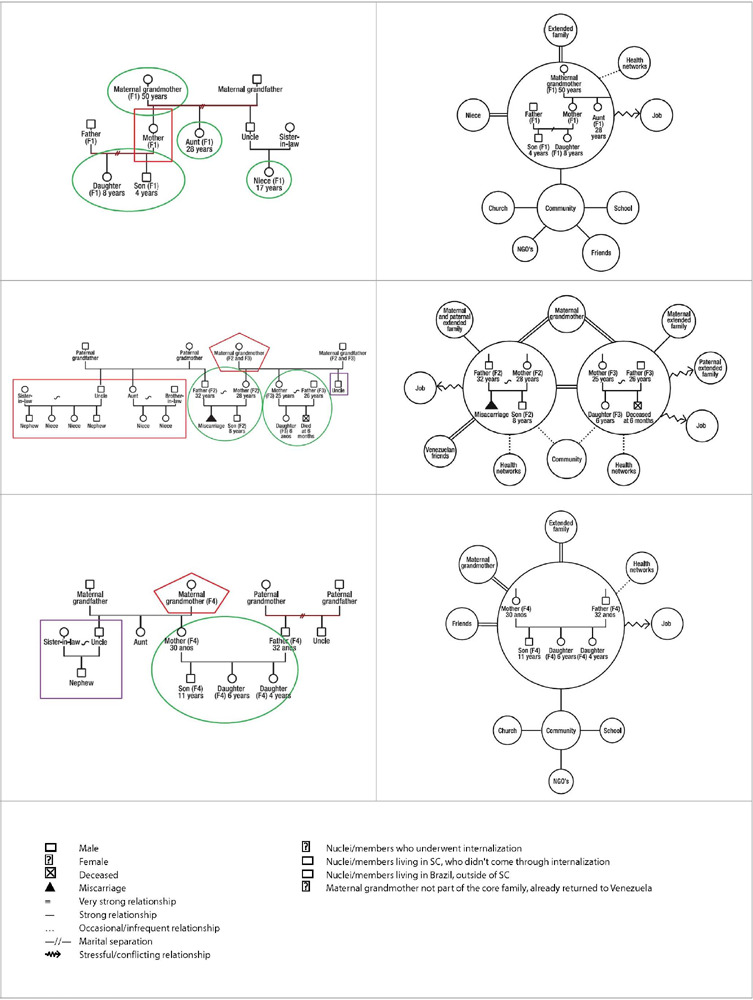
Genograms and ecomaps of the families participating in the study

In Chart 1, genograms and ecomaps constructed from the information of the four Venezuelan families are presented. These diagrams provide data characterizing the nuclei concerning the family development phase they were in at the time of migration. The life cycle phase of Venezuelan migrant families is mainly marked by the presence of young children. This characterization highlights the peculiarities of the migratory process with children and their care demands, as well as the presence of an informal support network, often represented by the grandmothers.

SC = Santa Catarina, Brazil.

Another notable point, illustrated in the genograms, is the identification of the migrant nuclei of the families. Migration flows vary, as indicated by the colors in the legend: nuclei currently residing in Santa Catarina due to the internalization process, nuclei in Santa Catarina but not internalized, and nuclei living in Brazil outside Santa Catarina.

In this context, it is clear that migrations are transitions that occur in different family nuclei throughout the life cycle.

Regarding the thematic category of migrants’ support networks, it is noted, from the ecomaps and interviews, the identification of four subcategories:

1. Family Network
2. Friends/Acquaintances Network
3. Community Network (work/Non-Governmental Organizations/Churches/schools)
4. Health Networks.

Among the interviewed families, the primary support network is established by the direct connection of the members themselves, regardless of whether they migrated simultaneously from Venezuela to Brazil. This network provides emotional security during the migration journey and in the adaptation periods after arrival at their destinations. The support from the family network is not only directed towards the immediate nucleus but also the extended family living in another state or country. Within the migrant family core, the maternal grandmother plays a fundamental role in daily domestic activities, in education, in the direct care of grandchildren, and is also respected for transmitting values, health cultural practices, and maintaining religious rituals.

*The mother is responsible for everything related to the children: making breakfast, lunch, giving them baths or assisting with baths - they do bathe themselves, but they still need help.* [...] *And she has to make lunch, sit with them* [the child]*. So, primarily, the one who feels the most stress at home is the mother.* (child’s grandmother, AF1)

In some migration experiences of maternal grandmothers, whose significant family function is reflected in adapting to the new life stage in another country, stories emerge of a void in integration and socialization of the elderly with the community. Coincidentally, during the research period, in two families, maternal grandmothers chose to return to Venezuela; in another, there was a report of a grandmother who also returned to her home country:

*One of the things my mother wishes for is to leave, because she wants to return to Venezuela. She says, ‘In Venezuela, I went out, worked, I found something to do. But here, I don’t know anyone, I don’t understand, I can’t speak the language. I want to go out but can’t because I don’t know anything.* (MF3)

The “extended” family network provides less recurrent support, yet its members seem to play a crucial role in financial aid for the continuity of the migrating nuclei’s transition:

*His aunt who lived here told us there was a job and that we’d earn, I don’t remember, maybe 2,000 reais a month back in 2016.* [...] *So, we said: let’s go to Brazil and after six months we’ll return.* (MF4)

The couple’s cohesion appears as a significant support factor in the family nucleus during the migratory journey. In one of the families, the adversities encountered strengthened the marital relationship:

*You know that partner you have, who went through a lot of hardships with you, and yet we became even closer. And here we are, right?* (MF2)

*My husband is the one who brings us peace and security. We feel calm and at peace with him around.* (MF4)

On the other hand, setbacks in the migration process signal frustration, as in the case of family F1, single-parent, where both adult daughters faced marital separations. In this scenario, the separation impacts the support network for the care of the children, requiring them to perform family roles and tasks independently:

*My daughter is a very happy child, even though she misses her father a lot. He is present in their lives, only through messages and audio. But not economically.* (AF1)

In this single-parent context, there is observed solidarity between the adult sisters concerning financial support, care for the children, and maintenance of the grandmother. The older sister, being the family’s head, began the migration on her own and was responsible for subsequently bringing other family members: the younger sister, the grandmother, the older sister’s children, and a niece, daughter of a brother residing in Ecuador:

*I can’t just abandon the children like that. After all, they call me ‘mother.’ So, I receive love and affection from them as a mother, so I feel responsible.* (AF1)

While the family network emerges as the primary psychosocial support, the network of friends and acquaintances is intrinsically linked to the migration project. They establish contacts with previously stabilized individuals and, based on comments about the characteristics of states and municipalities, decide to migrate. Upon arrival, they are welcomed and assisted by these networks:

*I hadn’t decided to come to Santa Catarina, but then I met a friend. She showed me photos, and the photos also conveyed that it’s much better here, very peaceful, secure, and so, I became interested until I decided to come.* (MF4)

Although this network proves fundamental in the migration process, some situations of helplessness create vulnerability, especially upon arrival at the destination. Here, misunderstandings between known migrants might occur, or even frustrations when their expectations are not met at the chosen location:

*The guy who was supposed to receive me here told me, ‘you have to arrive by Friday because I’m going to Venezuela. If you don’t get here by Friday, you’ll have nowhere to sleep because I want to leave you with my son here.’ I said, ‘okay.’ But there was a delay until Saturday* [...] *but his son never showed up. I wrote, wrote, and wrote, but he never appeared. We arrived on Saturday and he appeared on Tuesday.* (MF2)

The restructuring of life in Santa Catarina necessitates seeking local personal contacts, accessing social amenities, and establishing a community network. This network mainly consists of support received from workplaces, churches, schools, and NonGovernmental Organizations (NGOs). NGOs offer vital assistance in the early days following families’ arrivals, aiding in shaping a tangible sense of local settlement. This help can take the form of furniture donations, housing rentals, job offer information, or general support:

*Then the pastor said, ‘Who here has family?’ A lot of people said, ‘me, me.’ I cried, saying I didn’t have anyone. It was so cold. Then the pastor said, ‘I’ll pay for 3 people to stay in a hotel, but by noon tomorrow, you have to leave.* (MF2)

For children, parents believe the most significant network fostering integration is the school system. The role of teachers and peers in integrating the new culture can mitigate difficulties during the migration process, particularly when families transition between various places or cities:

*You see, she studies at a school and a teacher from there helps with transportation. The teacher drops her off here and takes a little longer to leave because she has to wait for the teacher.* (AF1)

However, instances of xenophobia have been reported, even within schools, as recounted by one of the families:

*We felt sorry for her because on Indigenous People’s Day, they congratulated her. They said, ‘Congratulations!’ and she responded, ‘Huh? But it’s not my birthday.’ They said, ‘No, but it’s Indigenous People’s Day.* (AF1)

Venezuelan families access health services in multiple ways, often prompted by specific needs such as pregnancy check-ups, illnesses, or emergencies that demand medical attention. Their connection with health centers tends to be tenuous. The primary entry point for public services is typically the Emergency Care Unit or the urgent/emergency departments of hospitals. During the pandemic, every family reported that at least one member had contracted the coronavirus:

*I think I’ve already told you that we went through COVID. We all got it, and I was hospitalized. I even ended up in the ICU, but my mother didn’t want to go to the hospital.* (AF1)

Some individuals, upon contracting Covid-19, opted against seeking medical attention, either out of fear of hospitalization and the ensuing separation from their families or due to potential language barriers. Language stands out as an obstacle when considering health services, even under different care circumstances. Yet, interviewees emphasize that the overall experience of accessing health services is often shaped by the warmth and competence of the professional reception. However, managing to get to the service, particularly given work commitments, can pose challenges:

*I thought that everyone who went to the hospital had to be in the ICU, so I was like, ‘What about the kids?’ You know? I was really unwell, but first and foremost, I thank God. They also took care of me. She* [girl] *is 7 years old and he* [boy] *is 4, but they would come into the room, onto the bed, every so often.* (AF1)

*My sister had to take insulin because she has... what do you call it... diabetes. She hasn’t yet addressed that aspect. She has other issues too; she had surgery. I also need to see a doctor, but I kept postponing, kept postponing it.* (AF1)

*There’s a doctor here, who is Colombian. He’s the one who sees me, right? He gives me a discount because I’m a foreigner, and he took it as a partnership since he’s also a foreigner.* (MF2)

As families recount their experiences in various places throughout their migratory path, they observe enhancements in health service accessibility. They note these improvements even without having a consistent health team to cater to their needs. Part of this perception is due to the constant change of residence, as they seek living conditions that better suit their economic standing:

*I changed health centers many times because we kept moving. I first went to Coqueiros and then, finally, I finished my prenatal care here in Areias, São José.* (MF3)

## DISCUSSION

The presence of a robust social and emotional support network is associated with violence prevention and skill enhancement. The efficacy of this network is evident from responses that indicate a significant reduction in symptoms, such as depression and feelings of helplessness. In the absence of this network, there is an observed increase in individuals’ vulnerability to risk situations, such as in forced migration flows^(15)^.

Migration, in itself, is a community phenomenon where the informal network organizes for an individual to migrate. Often, one might migrate alone, sometimes in groups, and throughout the journey, they establish their support networks^(16)^. The stability and strength of these networks influence how the migration process will unfold, which isn’t always dictated by financial factors but by the contexts of the networks already established at the destination. These are seen as support during the initial days of arrival^(17, 18)^, a sentiment explained by a sense of cultural and linguistic belonging and the shared experience of the migrant group. This reflects in a mutual understanding of the hardships faced during the journey^(17, 18)^. One probable reason for the increase in internalizations is the “Social Reunion” option.

Regarding the presence of elderly grandparents in families, while they ensure domestic care for children and informal support for couples to work outside, results showed a lack of social contacts outside the household. This situation worsened during the pandemic, with the elderly being considered a high-risk group for COVID-19. Although immigrant seniors travel with their descendants, which could reduce social isolation, factors such as family structure rearrangements and cultural differences can lead to generational conflicts. A feeling of lost social role, combined with a lack of proficiency in the host country’s language and the duration of stay, contributes to the isolation of this family member^(19)^.

This outcome highlights how forced migration, being an unplanned stage of the family life cycle, modifies roles and tasks within families. In the case of grandmothers, they might neglect aspects of their health, leisure routines, and even integration into the new country by focusing solely on raising their grandchildren.

For children, the most accessible formal network remains the school, which also serves as a space for family integration. However, it still presents challenges, such as difficulty with the new language and xenophobia. Based on this study, reinforcing this network can transform schools into a safer environment that promotes social empowerment, mitigating initial feelings of helplessness and aiding in mobilizing internal coping resources. In this regard, schools can also be an entry point for health services.

Concerning work activity, even participants with complete or incomplete higher education who were employed at the time of the survey, their current occupation does not match their field of study. This is observed even for the aunt of family 1 (AF1), who has extensive experience in human resources. One possible explanation is that migrants with higher educational levels find jobs more easily but in areas different from their training^(8, 9)^.

Based on the results, formal networks, including health and social services, were not the first sought-after resource. This finding may be related to a lack of trust, as in this study, these services are only sought in emergency situations.

Regarding the reasons for seeking care at Emergency Care Unit and hospital emergency/urgent care, we can point out: prioritizing work over health care; a lack of sensitive treatment regarding each migrant family’s sociocultural issues, and communication difficulties leading to fears of not being understood and/or welcomed as an immigrant. The mentioned reasons might be linked to difficulties in accessing and establishing ties in the primary healthcare centers’ services^(3, 4, 5, 9)^.

Considering the fragility of formal social networks, it is advisable to propose discussion spaces between immigrant community leaders and health teams. The involvement of community leaders, health professionals, and local decision-making agents can help clarify demands and develop service improvement proposals, aiming to assist in the social integration of immigrants.

In healthcare institutions, user participation is driven by greater democratization of information; by professionals recognizing users as subjects in the health care process and not just as objects of practices and prescriptions; and by making users aware of their rights and their role in defending their own interests^(20)^.

The participation of immigrants in health actions and services requires empowerment about their position and rights. For this, bonding with teams, grounded in trust and mutual dialogue, are ways to strengthen and recognize this network as social support in destination locations.

To include migration in community plans aimed at development and the realization of a more integrated society, it is essential to understand elements of family and social composition, develop local migration profiles, and conduct community mapping exercises to facilitate the process. This will prevent both receiving communities and immigrants from being harmed.

The 2030 Agenda for Sustainable Development recognizes migration as a driver of sustainable development. However, the relationship between migration and development is complex, involving political processes and the economic and social conditions of potential destination countries^(21)^.

### Study Limitations

This study has limitations stemming from data collection in the context of the COVID-19 pandemic, affecting both the researchers’ work and the availability of families to participate.

### Contributions to the Field

From the results found in this research, it is hoped to shed light on issues related to families in migration flows. The wish is for more actions to strengthen formal support networks, which include health and social services, to be discussed. With a global interdisciplinary perspective, this study also contributes to the understanding of some of the Sustainable Development Goals (SDGs), including health and well-being (SDG 3), reducing inequalities (SDG 10), and decent work and economic growth (SDG 8).

## FINAL CONSIDERATIONS

This study allowed for an understanding of the support networks of Venezuelan immigrant families who participated in the Interiorization Program and settled in the cities of Greater Florianópolis. It was observed that the primary informal support network is the nuclear family, and among formal networks, schools, churches, and civil society stand out.

Regarding the life cycle phase, all families are in the stage considered as families with young children. The grandmother is primarily responsible for the daily care of the children and the house, even with her own support network being limited. The situation of this family member in the immigrant family underscores the need for research focused on this profile.

Although the efforts of formal networks are commendable, there are noticeable gaps in reception and integration, including access to Primary Health Care services, as this is indicated as one of the weakest.
